# Complete genome sequence of *Brucella pseudintermedia* ASAG-D25, isolated from the wheat ear

**DOI:** 10.1128/MRA.01300-22

**Published:** 2023-08-03

**Authors:** Nanxi Wang, Yang Wang, Yi Jin, Baoyuan Guo, Hua Cui, Yongtan Yang

**Affiliations:** 1 Academy of National Food and Strategic Reserves Administration, Beijing, China; 2 College of Food Science and Engineering, Jilin Agricultural University, Changchun, China; University of Maryland School of Medicine, Baltimore, Maryland, USA

**Keywords:** *Ochrobactrum pseudintermedium*, *Brucella pseudintermedia*, wheat ear

## Abstract

*Brucella pseudintermedia* ASAG-D25 was isolated from the wheat ear sample in Xuzhou City, Jiangsu Province, China. The complete genome sequence of *B. pseudintermedia* will provide an important resource for better understanding of the genetic features of the species within the family of *Brucellaceae*.

## ANNOUNCEMENT

The genus *Brucella* includes Gram-negative, aerobic bacteria that do not produce hemolysis on blood agar (1) that were previously called *Ochrobactrum* spp. (2, 3). The complete genomic sequence of *B. pseudintermedia* ASAG-D25 will provide an important resource for better understanding of the genetic features of the species within the family of *Brucellaceae*.

The strain ASAG-D25 was isolated from the wheat ear sample collected in Xuzhou City, Jiangsu Province, China, by enrichment in minimal salt media with deoxynivalenol as the carbon source at 30°C in 2019. ASAG-D25 was grown aerobically at 30°C in Lysogeny broth for 15 h and collected by centrifugation at 4°C for 10 min for high-quality genomic DNA extraction using QIAGEN Genome Tip (Qiagen, Inc., Hilden, Germany) as per manufacturer’s recommendations. Whole-genome sequencing was carried out by Biomarker Technology, Inc. (Beijing, China) with a combination of the Nanopore PromethION 48 system and the Illumina NovaSeq 6000 platform from the same DNA extraction. Fragments were generated by shearing the genomic DNA using g-TUBE (Covaris, Woburn, MA, USA) without DNA size selection. The sheared DNA was used to construct an ONT Library using Ligation Sequencing Kit 1D (SQK-LSK109, ONT) with Native Barcoding Expansion 1–12 and 13–24. The nanopore base calling was performed using Guppy (version 3.2.6, ONT) with the parameter “–flowcell FLO-PRO002,” and 1,211,241,714 bp of raw data was generated, with an average read length of 9,110 bp. Short reads (<2,000 bp) and low-quality reads (quality scores < 6) were filtered by Filtlong (version 0.2.0) (4) and *de novo* assembly of the filtered reads (109,637 reads), with an average length of 9,390 bp and N_50_ value of 15,505 bp, resulted in an initial assembly (4,354,078  bp) using Canu (version 1.5) (5) and polished using Racon (version 1.4.3) (6). The tentative contigs were circularized and rotated to dnaA with Circlator (version 1.5.5) (7). The 350 bp library was constructed using Hieff NGS OnePot Pro DNA Library Prep Kit (Yeason, Shanghai, China) and sequenced on the Illumina NovaSeq 6000 platform. A total of 2,974,832 sequenced read pairs, with an average length of 147 bp, were checked for quality using FastQC (version 0.11.5) (8) and filtered using Trimmomatic (version 0.33) (9). The remaining 2,333,048 read pairs were mapped to the assembly with the Burrows-Wheeler Aligner (version 0.7.17) (10) for sequence and assembly error correction with Pilon (version 1.2.2) (11). Using BUSCO (version 5.1.2) with the alphaproteobacteria_odb10 database, the genome sequence is predicted to be 99.77% complete (12, 13). Default settings were used for all software unless otherwise noted.

The genome of ASAG-D25 comprises two chromosomes of 2,487,296 bp and 1,812,460 bp, with an average GC content of 57.93%. Annotation predicted 4,002 coding genes, 12 rRNAs, 58 tRNAs, and 4 ncRNAs by the NCBI PGAP (version 6.1) (14, 15). Average nucleotide identity (ANI) analysis using the OrthoANIu (16) revealed that ASAG-D25 is phylogenetically related to *Brucella pseudintermedia* CCUG 34735 (GCA_008932435.1, 99.43%), which was above the boundary of 95% ANI for species delineation (17).

## Data Availability

The full genomic sequence of *B. pseudintermedia* ASAG-D25 has been deposited in NCBI/GenBank under BioProject number PRJNA853445 with GenBank accession numbers CP099967.1 and CP099968.1, BioSample number SAMN29388680, and SRA numbers SRR19891259 (PromethION) and SRR19891260 (Illumina).
